# Rosmarinic acid exerts anti-inflammatory effect and relieves oxidative stress via Nrf2 activation in carbon tetrachloride-induced liver damage

**DOI:** 10.29219/fnr.v66.8359

**Published:** 2022-11-18

**Authors:** Yue-hong Lu, Yue Hong, Tian-yang Zhang, You-xia Chen, Zhao-jun Wei, Chun-yan Gao

**Affiliations:** 1 College of Bioscience and Bioengineering, North Minzu University, Yinchuan, China; 2 School of Public Health, Dali University, Dali, China; 3 School of Food Science and Engineering, Hefei University of Technology, Hefei, China

**Keywords:** rosmarinic acid, hepatoprotective effect, Nrf2 pathway, oxidative stress, inflammation

## Abstract

**Background:**

Rosmarinic acid (RA) has biological and pharmaceutical properties and shows hepatoprotective potential. However, the hepatoprotective mechanism of RA needs to be further elucidated in vivo and in vitro.

**Objective:**

This study was aimed to evaluate the protective effect of RA on carbon tetrachloride (CCl_4_)-induced liver injury and elucidate the hepatoprotective mechanism of RA in vivo and in vitro.

**Design:**

In vivo, the mice were orally administrated with RA (10, 20, and 40 mg/kg bw) daily for 28 consecutive days, and 1% CCl_4_ (5 mL/kg bw, dissolved in peanut oil) was used to induce liver injury. In vitro, the big rat liver (BRL) hepatocytes were pretreated with RA (0.2, 0.4, and 0.8 mg/mL) for 3 h, and then the hepatocytes were treated with CC1_4_ (final concentration, 14 mM) for 3 h to induce cell injury. The related indexes, including hepatic function, oxidative stress, protein expression of nuclear-factor erythroid 2-related factor 2 (Nrf2) pathway, inflammation, histopathological change, hepatocyte apoptosis, and mitochondrial membrane potential, were evaluated.

**Results:**

Oral administration of RA to mice considerably decreased the CCl_4_-induced elevation of serum alanine aminotransferase (ALT), alkaline phosphatase (ALP), triacylglycerols (TG), total cholesterol (TC), total bilirubin (TBIL), hepatic reactive oxygen species (ROS), malondialdehyde (MDA), nitric oxide (NO), 8-hydroxydeoxyguanosine (8-OHdG), tumor necrosis factor-α (TNF-α), interleukin-6 (IL-6), and interleukin-8 (IL-8). RA also increased the levels of hepatic glutathione (GSH), superoxide dismutase (SOD), and catalase (CAT) and the protein expressions of Nrf2, quinine oxidoreductase (NQO1), and heme oxygenease-1 (HO-1). Histopathological examinations indicated that RA (20 and 40 mg/kg bw) alleviated the liver tissue injury induced by CCl_4_. Moreover, RA inhibited the hepatocyte apoptosis caused by CCl_4_ based on TUNEL assay. In vitro, RA pretreatment remarkably recovered the cell viability and reduced the CCl_4_-induced elevation of AST, ALT, lactate dehydrogenase (LDH), ROS, and 8-OHdG. Immunohistochemistry staining demonstrated that pretreatment with RA markedly inhibited the expression of IL-6, inducible nitric oxide synthase (iNOS), cyclooxygenase-2 (COX-2), and Caspase-3 in CCl_4_-treated hepatocytes. Additionally, RA pretreatment significantly decreased the elevation of mitochondrial membrane potential in CCl_4_-treated hepatocytes.

**Conclusions:**

RA exerted a protective effect against CCl_4_-induced liver injury in mice through activating Nrf2 signaling pathway, reducing antioxidant damage, suppressing inflammatory response, and inhibiting hepatocyte apoptosis. RA could attenuate BRL hepatocyte ROS production, DNA oxidative damage, inflammatory response, and apoptosis induced by CCl_4_ exposure.

## Popular scientific summary

The popular scientific summary of the manuscript is as follows:

- The hepatoprotective activity of RA in vivo and in vitro was evaluated.
- RA protected the CCl_4_-induced liver damage via antioxidative and anti-inflammatory effects.
- RA activated the Nrf2 signaling pathway, thereby reducing CCl_4_-induced hepatic injury in mice.

Liver diseases, including hepatitis, cirrhosis, fibrosis, and hepatocellular carcinoma, are the most common chronic diseases in the world, which have continuously risen to become one of the foremost causes of death and illness over the past several decades (1). Lots of factors like inadequate nutrition, viral infection, alcohol and drug dependency, xenobiotic exposure, and metabolic diseases have been involved in the development and further progression of liver diseases (2). According to the Global Burden of Disease (GBD) project, liver diseases have caused over 2 million deaths in 2010, accounting for approximately 4% of the total global deaths (3). Therefore, liver disease is becoming a considerable public health burden. It is urgently needed to take various strategies to comprehensively fight against liver diseases, such as diet/lifestyle modification, early diagnosis, prevention in advance, timely medical treatment, exploration of safe and effective medications, and so on.

Carbon tetrachloride (CCl_4_) is a chemical agent, which is frequently used in the liver injury model. CCl_4_ is activated by liver microsomal cytochromes to generate trichloromethyl radical (CCl_3_), which induces oxidative stress by binding to cellular molecules, thereby leading to cell damage, inflammation, and apoptosis (4). Plant phenolic compounds have been reported to ameliorate liver damage induced by oxidative stress and inflammation and prevent liver disease (5–8).

Rosmarinic acid (RA), a natural phenolic compound, is commonly found in plants of *Lamiaceae* and *Boraginaceae* families (9), among other plants. RA is an ester of caffeic acid and 3,4-dihydroxyphenyl lactic acid (10, 11), corresponding to the hydroxycinnamic acid family. Structurally, it contains two catechol groups, which produce many biological and pharmacological activities, such as antioxidant (12), anti-inflammatory (12, 13), antitumoral (10), neuroprotective (10, 11), wound healing (14), hepatoprotective (15–18), among others. As far as the hepatoprotection of RA is concerned, the hepatoprotective mechanism needs to be further elucidated from the levels of molecules and cells.

In the present study, we aimed to evaluate the hepatoprotective effects of RA against CCl_4_-induced liver injury in vivo and in vitro, from the aspect of hepatic function, oxidative stress, protein expression of nuclear-factor erythroid 2-related factor 2 (Nrf2) signaling pathway, inflammation, histopathological change, hepatocyte apoptosis, and mitochondrial membrane potential.

## Materials and methods

### Chemicals

RA (≥97%, Mw 360.31 g/mol) was purchased from Aladdin (Shanghai, China). The diagnostic kits for superoxide dismutase (SOD), glutathione (GSH), catalase (CAT), malondialdehyde (MDA), 8-hydroxydeoxyguanosine (8-OHdG), reactive oxygen species (ROS), NO, interleukin-8 (IL-8), interleukin-6 (IL-6), tumor necrosis factor-α (TNF-α), and mitochondrial membrane potential assay were provided by the Nanjing Jiancheng Bioengineering Institute (Nanjing, China). Rabbit anti-cyclooxygenase-2 (COX-2), rabbit anti-caspase-3, rabbit anti-inducible nitric oxide synthase (iNOS) antibodies, rabbit anti-IL-6, DAPI, and Tunel kit were purchased from Servicebio Chemical Co. (Wuhan, China). Nrf2, OH-1, and quinine oxidoreductase (NQO1) antibodies were provided by Cell Signaling Technology (CST), (USA). Horseradish Peroxidase (HRP), goat anti-rabbit IgG was provided by Kirkegaard & Perry Laboratories (KPL), (USA). BRL hepatocyte was purchased from the Institutes for Biological Sciences Cell Resource Center (Shanghai, China).

### Determination of hepatoprotective effect in vivo

#### Animals and experimental design

Fifty male Kunming mice [18–22 g, license number of the experimental animals: SCXK (Xiang) 2019-0004] were provided by the Experimental Animal Centre of Dali University. All animal operations complied with the guidelines of Chinese Council for Animal Care. After adaptation to the environment for 7 days, the mice were assigned to five groups with 10 mice in each group. Mice were housed at temperature (22°C ± 1°C) and humidity (50–60%) with a 12 h light-dark cycle. Mice were fed to a standard pelleted diet. The normal and model groups (CCl_4_-treated) were administered physiological saline daily. The ideal dose of RA was established according to the preliminary experiment. As a result, the low, medium, and high-dosage RA-treated groups supplemented with RA at 10, 20, and 40 mg/kg bw daily for 28 consecutive days, respectively. On the 29th day, all the groups except the normal group administered 1% CCl_4_ (5 mL/kg bw, dissolved in peanut oil), while the normal group received peanut oil alone. All animals were administered by gavage. 24 h after the final CCl_4_,treatment animals were sacrificed. Serum was obtained by centrifugation blood samples at 3,000 rpm for 10 min at 4°C and then conserved at −20°C for further detection. The liver of each mouse was carefully dissected and immediately washed with ice cold saline and stored at −80°C for further analysis. The following equations were adopted to calculate the liver and spleen index: liver index = liver weight (mg)/body weight (g) and spleen index = spleen weight (mg)/body weight (g).

#### Determination of serum biochemical indexes

The serum levels of serum alanine aminotransferase (ALT), aspartate aminotransferase (AST), alkaline phosphatase (ALP), total cholesterol (TC), triacylglycerols (TG), and total bilirubin (TBIL) were assessed using a clinical automatic biochemical analyzer (Hitachi 7600-110, Japan), and the results were exhibited in U/L for ALT, AST, and ALP, μmol/L for TBIL, and mmol/L for TG and TC.

#### Determination of hepatic biochemical indexes

Liver was mixed with frozen normal saline (1:9, w:v) and then homogenized to prepare the liver homogenate 10.0% (w/v). The liver homogenate was centrifuged at 3,000 rpm for 10 min. Then, the homogenate supernatant was used for the determination of MDA, ROS, NO, 8-OHdG, SOD, CAT, GSH, TNF-α, IL-6, and IL-8. All these metrics were measured according to the kit instructions, and the results were exhibited in ng/mg protein (prot) for 8-OHdG, fluorescence/g prot for ROS, nmol/mg prot for MDA, μmol/g prot for NO and GSH, U/mg prot for CAT and SOD, and ng/g prot for TNF-α, IL-6, and IL-8*.* Additionally, the fluorescence images were also captured using a fluorescence microscope (Olympus, BX53) to reflect the ROS level.

#### Histopathological assessment of liver damage

The fixation of liver tissue was done by putting liver samples in 10% neutral buffered formalin for 24 h. After dehydration by ethanol and embedding in paraffin, the paraffin masses were cut into 5 μm sections. Finally, hematoxylin-eosin (H&E) was used to stain for the observation of histopathological changes.

#### Western blot analysis of Nrf2, HO-1, and NQO1

The livers were lysed and centrifuged to gain total protein extracts. The bicinchoninic acid (BCA) protein assay kit (Servicebio, China) was used to measure protein concentrations. After separation by electro phoresis on 10% sodium dodecyl sulfate polyacrylamide gel electrophoresis (SDS-PAGE) gels and transmembrane onto polyvinylidene difluoride (PVDF) membrane, the samples were blocked with 10% skimmed milk. Then, membranes were incubated with primary antibodies at 4°C for 12 h. Subsequently, membranes were washed and then incubated with secondary antibodies at room temperature for 30 min followed by visualization using the Electrochemiluminescence system. Representative blots were chosen from three independent experiments. Optical density value of western blots was performed with the use of Alpha software. Protein levels were normalized against those of corresponding β-actin.

#### TUNEL assay

Paraffin sections of control, model, and RA (40 mg/kg bw) pretreatment groups were used to carry out the TUNEL assay based on the kit instructions. Briefly, the paraffin sections were washed with xylene (three times, each for 10 min), absolute alcohol (three times, each for 15 min), and distilled water, in turn, to dewax. After the incubation with Proteinase K solution at 37°C for 20 min, the liver tissues were covered using 0.1% triton at room temperature for 20 min. The sections were dried, and the reaction solution (TDT:dUTP:buffer, 1:5:50) was added to cover the tissues. Then, the sections were put in a moist box and incubated at 37°C for 2 h. After that, the nucleus was counterstained with dihydrochloride (DAPI). Finally, the treated slides were sealed with anti-fade mounting medium. Images were obtained using a fluorescence microscope (400×). Ten microscopic fields in each group were randomly selected for the quantitative analysis.

### Determination of hepatoprotective effect in vitro

#### Determination of cell viability

BRL hepatocytes were seeded onto 96-well plates at 2.5 × 10^3^ cells per well. After incubation for 12 h, cells of RA pretreatment groups were treated with 150 μL of RA (0.8, 0.4, and 0.2 mg/mL in cell-culture medium) for 4 h. The control and model groups were given 150 μL of cell-culture medium. Then, 40 μL PBS was added to the control group, while the other groups were treated with 40 μL CC1_4_ (100 mM) for 3 h to induce cell injury. Finally, microculture tetrazolium assay was used to evaluate cell viability. The results were expressed as percent cell viability.

#### Determination of the AST, ALT, and LDH activities in supernatants

BRL hepatocytes were seeded into 24-well plates at 2.5×10^4^ cells per well. After incubation for 24 h, cells of RA pretreatment groups were treated with 750 μL of RA (0.8, 0.4, and 0.2 mg/mL in cell-culture medium) for 4 h. The control and model groups were given 750 μL of cell-culture medium. Then, 200 μL PBS was added to the control group, while the other groups were treated with 200 μL CC1_4_ (100 mM) for 3 h to induce cell injury. Finally, the AST, ALT, and LDH activities in supernatants were determined using a clinical automatic biochemical analyzer (Hitachi 7180, Japan).

#### Determination of the ROS and 8-OHdG

The BRL hepatocytes were digested by pancreatin. Then, the homogenates were centrifuged to get cell precipitate. Finally, the levels of ROS and 8-OHdG were determined according to the kit instructions.

#### Immunohistochemical analysis for IL-6, iNOS, COX-2, and Caspase-3

The cell climbing pieces were prepared, and the cells were fixed in acetone for 15 min. Then, 3% bovine serum (BSA) was used to cover the cells for 30 min. The treated slides were incubated with IL-6 (1:800), COX-2 (1:500), Caspase-3 (1:200), and iNOS (1:500) antibodies in a moist box at 4°C for 12 h, followed by incubation with HRP labeled goat anti-rabbit IgG for 50 min. DAB was used in color development and counterstained by hematoxylin. The slides were rehydrated with different concentration gradients of alcohol (75, 85, and 100%) once for 5 min and sealed with neutral tree gum. Images were captured by a light microscopy (400×).

#### Determination of mitochondrial membrane potential (*∆*Ψ*m*)

A mitochondrial membrane potential assay kit with JC-1 was used to detect the change of ***∆***Ψ***m*** in the BRL hepatocytes. ***∆***Ψ***m*** was determined by the green and red fluorescence, and a confocal laser scanning microscopy (CLSM, TCS SP8, Leica, Germany) was used to capture the images.

### Statistical analysis

All the experiments were conducted in triplicates, and the experimental data were exhibited in mean ± standard deviation (SD). One-way analysis of variance (ANOVA) and Duncan’s multiple range test were conducted to analyze statistical significance between the means by SPSS (version 17.0). Spearman correlation was performed to analyze the correlation between antioxidant markers and liver damage-related indicators.

## Results

### Hepatoprotective effect of RA in vivo

#### Effect of RA on liver and spleen index

The administration of CCl_4_ dramatically elevated the liver and spleen index compared to the normal group (*P* < 0.05), indicating significant swelling of liver and spleen (Table 1). However, the CCl_4_-treated mice pretreated with RA at a high dosage (40 mg/kg bw) showed a marked decrease of liver and spleen index (*P* < 0.05) compared to the model group, indicating a preventive effect against CCl_4_-induced liver and spleen swelling in mice.

**Table 1:** Effect of RA pretreatment on the liver and spleen index in CCl_4_-treated mice ($\bar{X}$ ± SD, *n* = 10)

Groups	Liver index	Spleen index
Normal	35.59 ± 3.05^a^	1.68 ± 0.18^a^
Model	48.41 ± 3.79^b^	2.24 ± 0.43^b^
RA (10 mg/kg bw) + CCl_4_	48.26 ± 1.88^b^	2.13 ± 0.50^ab^
RA (20 mg/kg bw) + CCl_4_	47.25 ± 3.22^bc^	1.94 ± 0.36^ab^
RA (40 mg/kg bw) + CCl_4_	43.50 ± 2.37^c^	1.72 ± 0.22^a^

Values within a column with different letters indicate significant difference among different groups at *P* < 0.05.

#### Effect of RA on serum biochemical indexes

As shown in Table 2, CCl_4_-induced toxicity caused significant elevation in serum biochemical indexes, including ALT, AST, ALP, TBIL, TC, and TG. In contrast, the 4-week pretreatment of mice with RA reduced the biochemical parameters in a dose-dependent manner, particularly when the dosage was increased from 20 to 40 mg/kg bw.

**Table 2:** Effect of RA pretreatment on the levels of serum ALT, AST, ALP, TBIL, TC, and TG in CCl_4_-treated mice ($\bar{X}$ ± SD, *n* = 10)

Groups	ALT (U/L)	AST (U/L)	ALP (U/L)	TBIL (μmol/L)	TC (mmol/L)	TG (mmol/L)
Normal	30.91 ± 2.91^a^	119.91 ± 24.32^a^	181.45 ± 20.12^a^	2.19 ± 0.54^a^	1.33 ± 0.27^a^	0.22 ± 0.06^a^
Model	1965.20 ± 114.08^d^	2196.50 ± 244.67^d^	356.00 ± 19.48^d^	9.67 ± 1.10^d^	2.81 ± 0.15^c^	1.86 ± 0.20^d^
RA (10 mg/kg bw) + CCl_4_	1596.72 ± 164.94^c^	1773.82 ± 278.34^c^	323.50 ± 30.24^c^	8.87 ± 1.03^cd^	1.80 ± 0.15^b^	1.84 ± 0.20^d^
RA (20 mg/kg bw) + CCl_4_	1595.40 ± 116.20^c^	1570.20 ± 181.280^bc^	301.00 ± 29.75^c^	8.19 ± 1.01^bc^	1.62 ± 0.20^b^	1.41 ± 0.18^c^
RA (40 mg/kg bw) + CCl_4_	1370.00 ± 137.82^b^	1438.45 ± 3173.26^b^	254.73 ± 23.24^b^	7.52 ± 0.90^b^	1.30 ± 0.19^a^	0.75 ± 0.10^b^

Values within a column with different letters indicate significant difference among different groups at *P* < 0.05.

#### Effect of RA on oxidative damage indicators in liver tissue

**Fig. 1:**
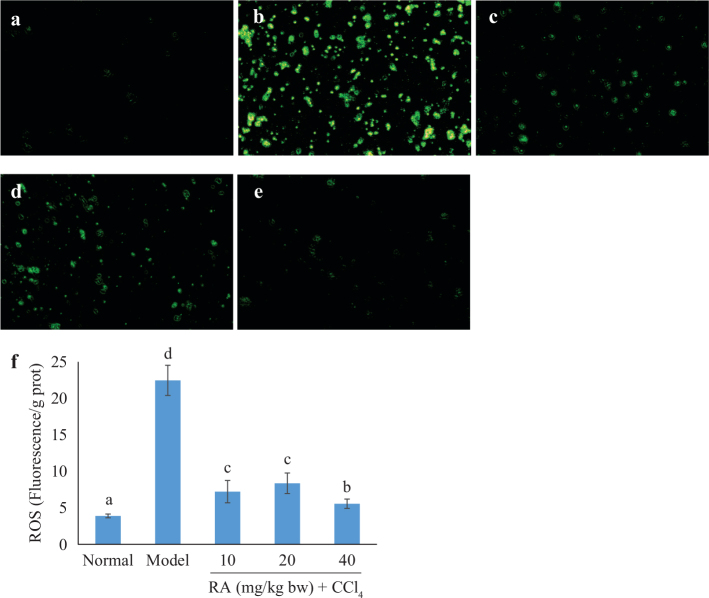
Effect of RA on ROS level (100×). (a) Normal group; (b) model group; (c–e) pretreated with RA at 10, 20, and 40 mg/kg bw before CCl_4_ treatment, respectively; (f) quantitative analysis of fluorescence images.

ROS, MDA, NO, and 8-OHdG were detected to evaluate the degree of oxidative damage in liver tissue. DCFH-DA (2, 7-dichlorofuorescin diacetate) was used as a fluorescence probe to detect the ROS level. In the normal group, the ROS level was 3.89 fluorescence/g prot. In the model group, the ROS level sharply increased to 22.46 fluorescence/g prot (*P* < 0.05). However, the CCl_4_-induced increase in ROS production was significantly inhibited by pretreatment with RA. The ROS level of low-, medium-, and high-dosage RA-treated groups was 7.22, 8.36, and 5.56 fluorescence/g prot, respectively (Fig. 1f). Furthermore, the fluorescence images also demonstrated that oral administration of CCl_4_ resulted in a significant increase in ROS production with strong green fluorescence (Fig. 1b). Consistently, oral supplementation of CCl_4_-treated mice with RA reduced the ROS production with weak green fluorescence (Fig. 1c–e). In accordance with the ROS results, CCl_4_-induced toxicity caused a 2.09-, 10.71-, and 0.96-fold increase of MDA, NO, and 8-OHdG than in the normal group, respectively (Table 3). In contrast, RA pretreatment dramatically diminished the levels of MDA, NO, and 8-OHdG. It was worth mentioning that no significant differences in MDA and 8-OHdG levels were observed between RA-pretreated and normal groups (*P* > 0.05).

**Table 3:** Effect of RA pretreatment on the levels of liver MDA, NO, 8-OHdG, SOD, CAT, and GSH in CCl_4_-treated mice ($\bar{X}$ ± SD, *n* = 10)

Groups	MDA (nmoL/mg prot)	8-OHdG (ng/mg prot)	NO (μmoL/g prot)	CAT (U/mg prot)	SOD (U/mg prot)	GSH (μmol/g prot)
Normal	1.30 ± 0.27^a^	2.31 ± 0.47^a^	0.07 ± 0.01^a^	111.91 ± 13.47^b^	181.89 ± 13.06^b^	6.61 ± 0.97^c^
Model	4.02 ± 0.88^b^	4.52 ± 0.60^b^	0.82 ± 0.10^d^	86.67 ± 7.43^a^	130.83 ± 9.91^a^	2.82 ± 0.47^a^
RA (10 mg/kg bw) + CCl_4_	1.47 ± 0.36^a^	2.63 ± 0.49^a^	0.70 ± 0.05^cd^	85.79 ± 17.04^a^	145.74 ± 13.58^a^	3.85 ± 0.98^b^
RA (20 mg/kg bw) + CCl_4_	1.38 ± 0.35^a^	2.72 ± 0.47^a^	0.62 ± 0.06^c^	82.30 ± 12.04^a^	147.26 ± 24.60^a^	2.83 ± 0.89^a^
RA (40 mg/kg bw) + CCl_4_	1.51 ± 0.21^a^	2.24 ± 0.36^a^	0.37 ± 0.03^b^	118.33 ± 13.60^b^	174.20 ± 14.46^b^	3.89 ± 0.50^b^

Values within a column with different letters indicate significant difference among different groups at *P* < 0.05.

#### Histopathological assessment of liver damage

**Fig. 2:**
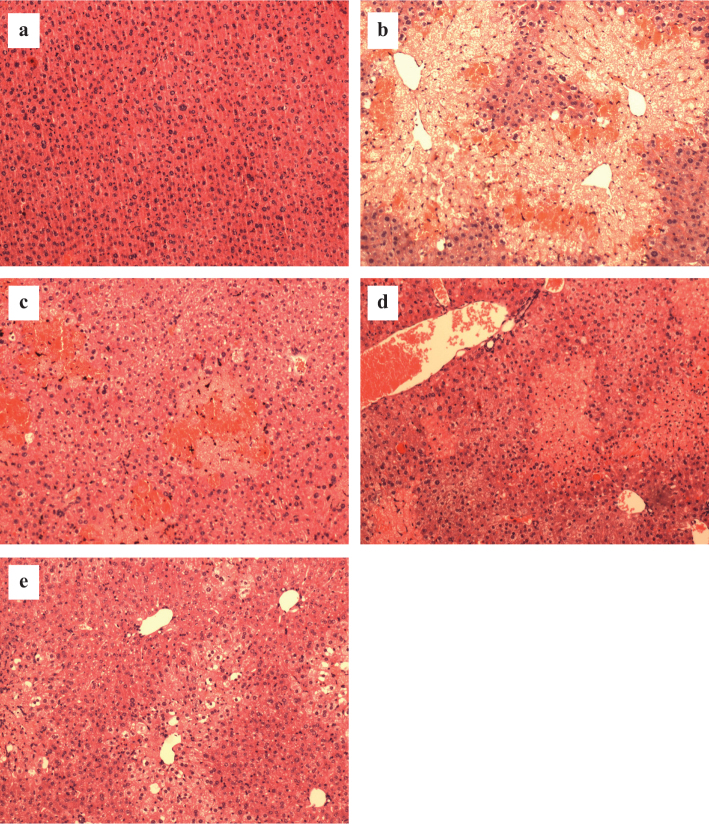
Images of histopathological examination (hematoxylin and eosin, 100×). (a) Normal group; (b) model group; (c–e) pretreated with RA at 10, 20, and 40 mg/kg bw before CCl_4_ treatment, respectively.

The hepatic tissues with intact cytoplasm, clear hepatic nucleus and nucleolus, and visible central vein were observed in the normal animals (Fig. 2a). Inversely, the histological examination revealed evidence of severe injury of liver tissues with prominent hepatocyte necrosis and steatosis, condensed nuclei, large amounts of inflammatory cells, and collapse of parenchyma in CCl_4_-injured animals (Fig. 2b). However, marked alleviation of liver tissue injury was observed in RA-pretreated group at the dose of 20 and 40 mg/kg bw (Fig. 2e).

#### Effect of RA on the activity of antioxidant enzymes

As shown in Table 3, the levels of liver CAT, SOD, and GSH in the model group were significantly lower than those of the normal group, which were 77.47, 71.93, and 42.66% of the normal group, respectively. Pretreatment of RA at a dosage of 40 mg/kg bw showed a significant improvement of these indexes, especially of CAT and SOD that was comparable to the normal group.

#### Effect of RA on Nrf2, HO-1, and NQO1 protein expression

**Fig. 3:**
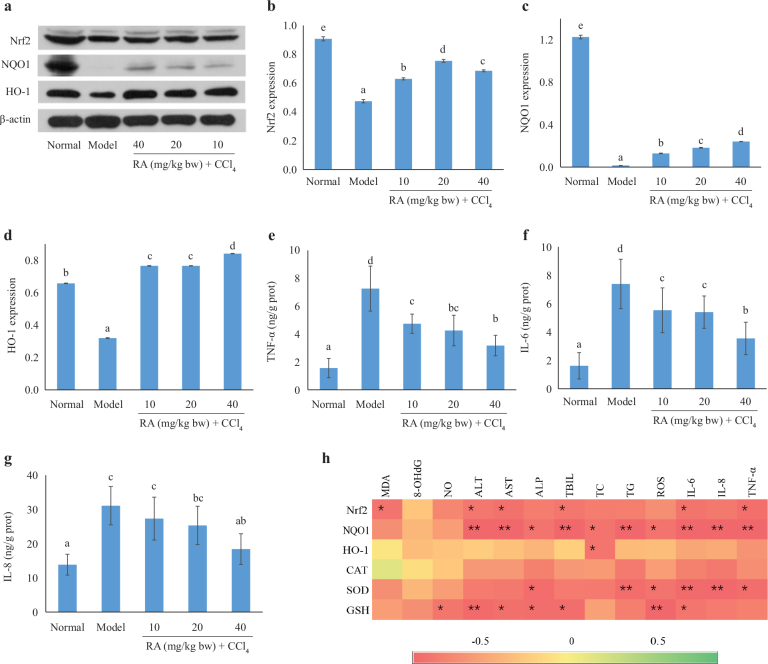
Effect of RA on Nrf2, HO-1, and NQO1 protein expressions (a–d) and the levels of TNF-α, IL-6, and IL-8 (e–g). Heatmap of correlation analysis between the antioxidant makers and liver damage-related indicators. The colors range from red (negative correlation) to green (positive correlation) (h).

To explore the antioxidant mechanism, the key proteins involved in Nrf2 signaling pathway were determined by western blotting (Fig. 3a–d). Downreguzlation was observed of the Nrf2, heme oxygenease-1 (HO-1), and NQO1 protein expression by the CCl_4_ treatment. In the presence of RA, dose-dependent upregulation was found in the NQO1 protein expression compared to the CCl_4_ treatment (Fig. 3c). In contrast, the upregulation effect observed for the Nrf2 and HO-1 was independent from the RA pretreatment dose (Fig. 3b and d).

#### Effect of RA on the levels of TNF-α, IL-6, and IL-8

CCl_4_ exposure resulted in drastic inflammatory response accompanied by a 3.65-, 3.56-, and 1.25-fold increase of hepatic TNF-α, IL-6, and IL-8 levels than in the normal group, respectively (Fig. 3e–g). In contrast, RA pretreatment significantly suppressed the increase in the levels of these indexes in a dose-dependent manner.

#### Correlation analysis between the antioxidant makers and liver damage-related indicators

As shown in Fig. 3h, generally, the antioxidant makers showed negative correlation with the liver damage-related indicators. Nrf2 was strongly negatively correlated with MDA, ALT, AST, TBIL, IL-6, and TNF-α. Except for MDA, NO, and 8-OHdG, a significant negative correlation was found between NQO1 and all the other liver damage-related indicators. The negative correlation of SOD with ALP, TG, ROS, IL-6, IL-8, and TNF-α was found. Significant negative correlation was existed between GSH and NO, ALT, AST, ALP, TBIL, ROS, and IL-6. However, HO-1 only had significant negative correlation with TC, and no significant correlation was observed between CAT and all liver damage-related indicators.

#### Effect of RA on hepatocyte apoptosis

**Fig. 4:**
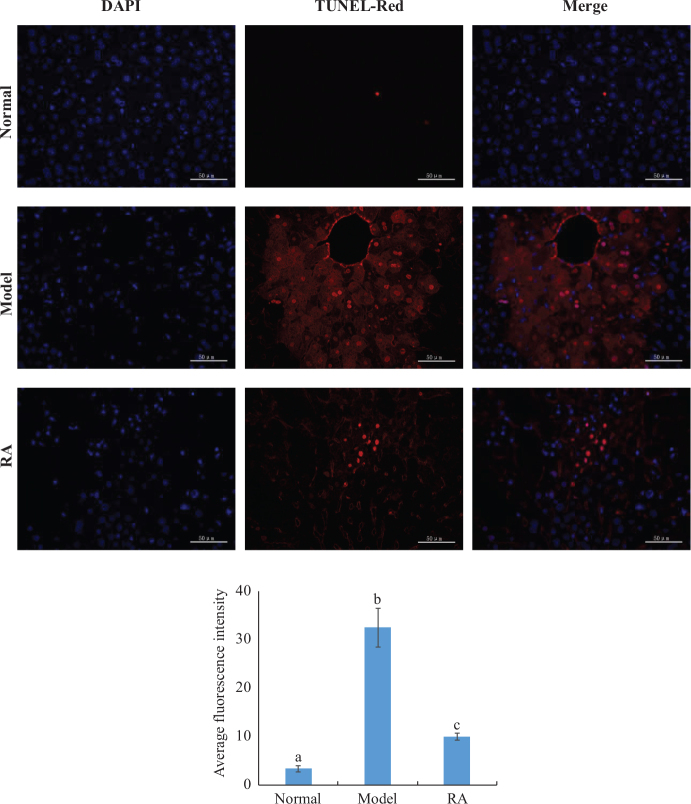
TUNEL assay images (400×) of normal, model, and RA (40 mg/kg bw) + CCl_4_ groups and statistical analysis of TUNEL assay images. Values expressed as mean ± SD in each group (*n* = 10). The different capital letters indicate significant differences among the different groups at *P* < 0.01.

TUNEL assays were performed to elucidate the effect of RA on hepatocyte apoptosis, and the results were exhibited in Fig. 4. Fluorescence observations showed considerable toxic effect of CCl_4_ on the liver tissues as reflected by severe apoptosis with enhanced red fluorescence. Interestingly, the liver tissues of RA-pretreated group with 40 mg/kg bw exhibited slight hepatocyte apoptosis with attenuated red fluorescence when compared with the model group.

Being consistent with the results of fluorescence images, the quantitative analysis results showed that CCl_4_ resulted in a 8.56-fold increase in hepatocyte apoptosis than in the normal group. RA pretreatment (40 mg/kg bw) decreased the level of hepatocyte apoptosis by 69.28% in comparison to the CCl_4_ treated group.

### Hepatoprotective effect of RA in vitro

#### Effect of RA on the cell viability and AST, ALT, and LDH activities

The results showed hepatoprotective effect of RA in a dose-dependent manner against CCl_4_-toxicity (Table 4). The cell viability of the model group was 24.33%, while the RA presented dose-dependent recovery with values in the range of 43.30–65.94%. The treatment of CCl_4_ considerably elevated the AST, ALT, and LDH activities compared to the normal control group (*P* < 0.05), indicating severe hepatocyte injury of CCl_4_ treatment. In contrast, pretreatment with RA markedly inhibited the rise of AST, ALT, and LDH activities induced by CCl_4_.

**Table 4:** Effect of RA pretreatment on cell viability and AST, ALT, and LDH activities of BRL hepatocyte injured by CC1_4_ ($\bar{X}$ ± SD, *n* = 5)

Groups	Cell viability (%)	AST (U/L)	ALT (U/L)	LDH (U/L)
Normal	93.13 ± 0.16^a^	5.17 ± 0.75^a^	1.67 ± 0.52^a^	81.83 ± 2.71^a^
Model	24.33 ± 0.94^b^	19.50 ± 1.05^b^	3.33 ± 0.52^b^	164.33 ± 2.42^b^
RA (0.2 mg/mL) + CCl_4_	43.30 ± 0.51^d^	17.17 ± 1.47^c^	3.00 ± 0.63^bc^	154.17 ± 3.43^c^
RA (0.4 mg/mL) + CCl_4_	54.91 ± 0.52^e^	15.50 ± 1.64^cd^	2.17 ± 0.41^ac^	147.50 ± 1.38^d^
RA (0.8 mg/mL) + CCl_4_	65.94 ± 0.73^f^	15.00 ± 1.26^d^	1.83 ± 0.98^a^	136.33 ± 2.66^e^

Values within a column with different letters indicate significant difference among different groups at *P* < 0.05.

#### Effect of RA on the levels of ROS and 8-OHdG in the hepatocyte

**Fig. 5:**
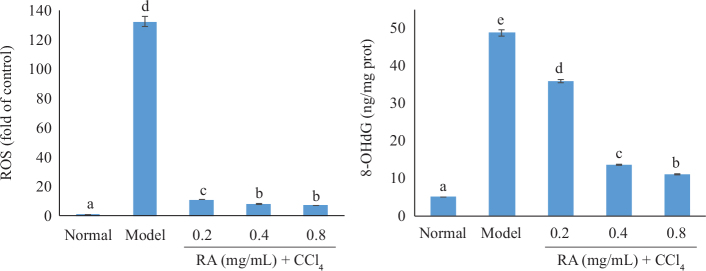
Effect of RA on the levels of ROS and 8-OHdG in BRL hepatocyte.

As exhibited in Fig. 5, CCl_4_-induced toxicity caused significant increase in the hepatocyte levels of ROS and 8-OHdG, which were 131.32 and 8.65 times higher than that of the normal cells, respectively. However, the RA exhibited a dose-dependent inhibition effect on the levels of ROS and 8-OHdG.

#### Effect of RA on the levels of Caspase-3, IL-6, COX-2, and iNOS in the hepatocyte

**Fig. 6:**
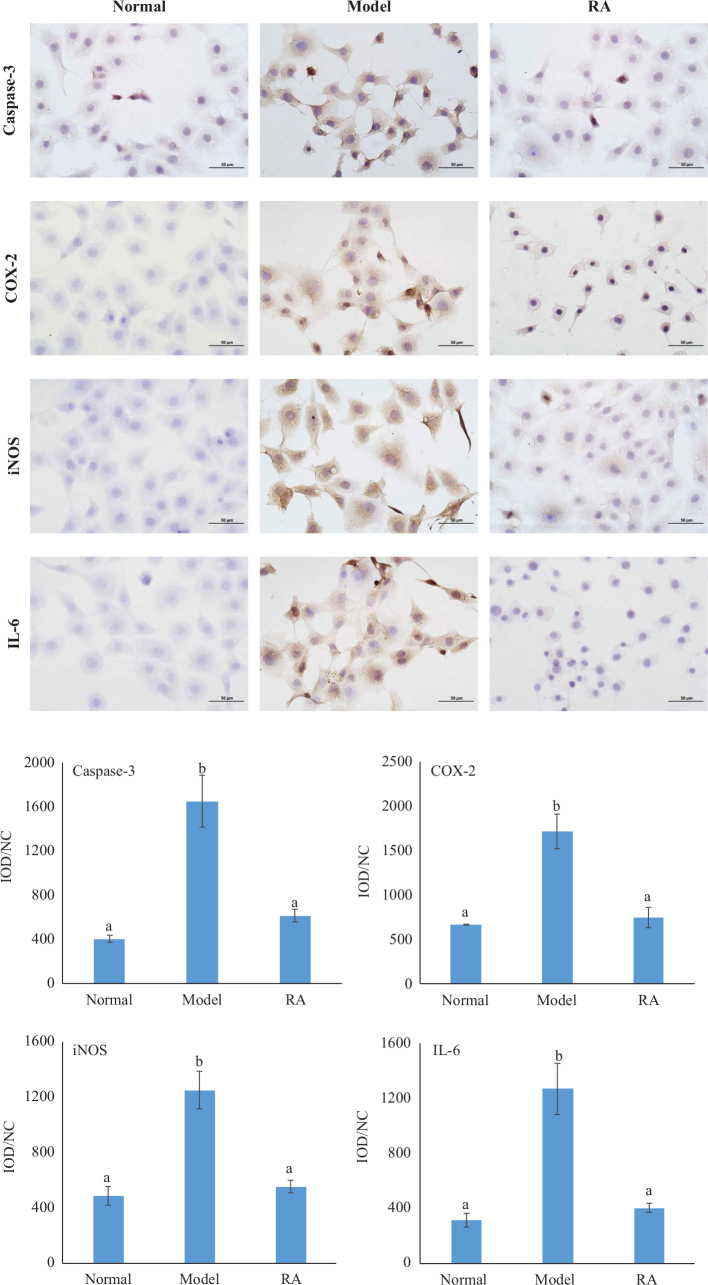
Effect of RA (0.8 mg/mL) on the levels of IL-6, COX-2, iNOS, and Caspase-3 with the immunohistochemical method (400×). Statistical analysis was based on the values of IOD/NC (integrated optical density/number of cells). Values expressed as mean ± SD in each group (*n* = 10). The different capital letters indicate significant differences among the different groups at *P* < 0.05.

Immunohistochemical staining analysis indicated that CCl_4_ treatment caused considerable increase in the expression of hepatocyte Caspase-3, IL-6, COX-2, and iNOS with brown staining (Fig. 6). In contrast, RA pretreatment (0.8 mg/mL) considerably suppressed the increase in the expression of these proteins with slight brown staining. The quantitative analysis results indicated that the expression of hepatocyte Caspase-3, IL-6, COX-2, and iNOS by the RA pretreatment was comparable to the normal hepatocytes (Fig. 6).

#### Effect of RA on the mitochondrial membrane potential (*∆*Ψ*m*)

**Fig. 7:**
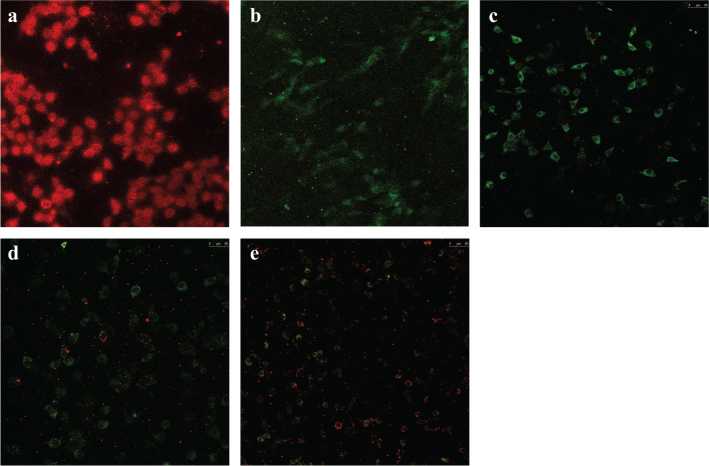
Effect of RA on the mitochondrial membrane potential. (a) Normal group; (b) model group; (c–e) pretreated with RA at 10, 20, and 40 mg/mL before CCl_4_ treatment, respectively.

Fluorescence observations revealed significant toxic effect of CCl_4_ on the mitochondria of BRL hepatocytes as reflected by altered *∆*Ψ*m*. As shown in Fig. 7, the normal hepatocytes possessed higher *∆*Ψ*m* with the exhibition of red fluorescence, indicating a healthy mitochondria. However, the CCl_4_-treated hepatocytes had lower *∆*Ψ*m* with the exhibition of green fluorescence, indicating a severe damaged mitochondria. RA considerably elevated the red fluorescence in a dose-dependent manner compared with the model group, which suggested that RA could protect against mitochondrial injury caused by CCl_4_.

## Discussion

It has been documented that CCl_4_ is converted to a trichloromethyl free radical (CCl_3_) and trichloromethyl peroxy radical (OOCCl_3_) by hepatic cellular cytochrome P450 in the liver (4). The free radicals produced during CCl_4_ poisoning attack on unsaturated fatty acids of phospholipids presenting in the cell membrane, in turn, leading to lipid peroxidation and production of oxygen-centered lipid radicals (LO· and LOO) (19). A marked increase of serum ALT, AST, ALP, TG, TC, and TBIL is the symbol of cellular leakage, alteration of transport functions and cellular wall integrity, and impairment of metabolic function of hepatocyte (20) during CCl_4_-induced liver damage. The results obtained revealed that RA could prevent CCl_4_-induced liver injury in mice. The in vitro experiment results also revealed that RA pretreatment markedly prevented the elevation of ALT, AST, and LDH activities in supernatants induced by CCl_4_, which further confirmed that RA could protect the liver from CCl_4_-induced damage.

The production of free radicals produced by CCl_4_ poisoning resulted in the increase of ROS. MDA, an indicator of lipid peroxidation, and its content reflects the extent of lipid peroxidation. NO is a highly reactive oxidant and a multifunctional free radical, which is produced by iNOS (21). Excessive NO could cause liver damage by producing ROS via a reaction with a superoxide anion (20). 8-OHdG, a sensitive index of DNA oxidative damage, derives from the ROS attacks on the deoxyguanine of DNA (22). The level of 8-OHdG reflects the degree of DNA oxidative damage and oxidative stress state. A marked increase in hepatic ROS, MDA, NO, and 8-OHdG level of model group comparison with the normal group suggested severe oxidative damage leading to tissue injury and failure of the antioxidant-defense mechanisms to scavenge excessive free radicals. Significant reductions of these indicators in the liver after the administration of RA were observed when compared with CCl_4_-treated mice, which indicated that the RA could protect the CCl_4_-induced liver injury by scavenging ROS and inhibiting lipid peroxidation, production of NO and DNA oxidative damage. Consistent with the in vivo results, the hepatocytes in CCl_4_-treated group experienced severe oxidative damage, and RA with strong antioxidant activity inhibited the production of ROS and DNA oxidative damage. Additionally, RA pretreatment at 20 and 40 mg/kg bw considerably alleviates the pathological alterations of liver tissue, which further confirmed the hepatoprotective effect of RA.

Antioxidant enzymes play an important role in preventing the CCl_4_-induced liver oxidative damage. The SOD, CAT, and GSH constitute a powerful antioxidant defense system to fight against oxidative damage. SOD catalyzes the binding of peroxyl radical (ROO·) to the proton to generate H_2_O_2_ and O_2_ (23). CAT converts H_2_O_2_ to H_2_O and O_2_ and suppresses the production of HO (24). GSH contributes greatly to the detoxification by binding to the free radicals, and the liver necrosis occurs when GSH reserve declines considerably (25). Changes in antioxidant enzyme activity reflect the ability of the liver to fight against oxidative stress induced by CCl_4_. Significant rises of SOD, CAT, and GSH levels in response to RA pretreatment (40 mg/kg bw) were observed when compared with the model group, suggesting that RA protects the CCl_4_-induced liver oxidative damage by elevation of antioxidant enzyme activity.

Nrf2, an important transcription factor, is closely related cellular anti-oxidative stress. HO-1, an important antioxidant enzyme, catalyzes prooxidant heme catabolism into ferrous iron, carbon monoxide, and bilirubin (26). NQO1, a cytosolic flavoenzyme, catalyzes the reduction of quinones to hydroquinones by a single-step two-electron reduction reaction (27). The activation of Nrf2 signaling pathway and the upregulation of downstream antioxidant/detoxifying enzymes such as HO-1, NQO1, SOD, GSR, and CAT play an important role in antioxidation and maintaining the cellular homeostasis (28). In the present study, the increases in Nrf2, HO-1, and NQO1 protein expressions in RA treatment groups compared with the model group revealed that RA could protect the CCl_4_-induced liver injury through activating the Nrf2 signaling pathway and upregulating the downstream antioxidant enzymes. Our result was consistent with the previous study that RA could promote the Nrf2 nucleus transport and then upregulate the expression of HO-1, NQO1, GCLC, and GCLM in lipopolysaccharide/D-galactosamine-induced acute liver injury in mice (16). The mitogen-activated protein kinase (MAPK) family, involving in a series of cell physiological activities such as cell growth, development, differentiation, and apoptosis, is mainly composed of three subfamilies, namely, extracellular-signal-regulated protein kinase (ERK), p38 MAPK, and JNK (c-Jun N-terminal kinase). It has been reported that the activation of Nrf2 signaling pathway was associated with MAPK family (28, 29). Next, a further study is necessary to reveal the mechanism of Nrf2 signal pathway activated by RA from the aspect of MAPK signal transduction pathway.

Hepatocyte damage is always accompanied by inflammatory response. ROS produced by CCl_4_ activates the innate immune system and Kupffer cells. Activated Kupffer cells release proinflammatory cytokines. TNF-α, as an inflammatory mediator produced in CCl_4_-inducedd liver injury, stimulates immune-related cells to produce many cytokines, such as IL-6, IL-8, and IL-1β (30). In turn, the inflammatory response is caused. Results obtained from this study revealed that RA treatment markedly inhibited the increase of hepatic TNF-α, IL-6, and IL-8 and BRL hepatocyte IL-6, COX-2, and iNOS caused by CCl_4_, which indicated that RA could suppress the inflammatory response to protect the liver damage. Results obtained of this study indicated that RA could suppress the inflammatory response by inhibiting the inflammatory cytokines. During inflammatory responses caused by CCl_4_, TNF-α receptor 1 (TNFR1) initiates TNF-α, which activates nuclear factor kappa B (NF-κB) (7). NF-κB plays a key role in inflammatory responses and immune responses. It has been reported that many phytochemicals protect against CCl_4_-induced liver damage in rats through the inhibition of NF-κB transactivation (16, 31, 32). Additionally, toll-like receptors (TLRs), a member of the pattern recognition receptor family, also involve in the activation of inflammatory responses (32, 33). Therefore, the activation of NF-κB and TLRs signaling pathway may be involved in the suppression of inflammatory response of RA.

The correlation analysis between antioxidant makers (SOD, CAT, GSH, Nrf2, OH-1, and NQO1) and liver damage-related indicators (ALT, AST, ALP, TG, TC, TBIL, ROS, MDA, NO, 8-OHdG, TNF-α, IL-6, and IL-8) was performed. The divergences in correlations between different antioxidant makers and various liver damage-related indicators could be attributed to the divergence in antioxidant mechanism of the individual antioxidant maker. For examples, HO-1 catalyzes substrate into ferrous iron, CO, and bilirubin, which play a key role in antioxidant and anti-inflammatory (26), whereas NQO1 plays an antioxidant role through exerting cytophylaxis and removing superoxide (34).

TUNEL assay results indicated that RA could inhibit the hepatocyte apoptosis induced by CCl_4_ in mice. Caspase-3, an important member of the apoptotic protease family, plays an important role in cell apoptosis, and its level could reflect the degree of apoptosis (35). In vitro results indicated that the RA could suppress the elevation of Caspase-3 level induced by CCl_4_, suggesting the inhibition of hepatocyte apoptosis, which was in consistent with the result of TUNEL assay.

Free radicals and lipid peroxidation produced during CCl_4_ poisoning cause mitochondrial DNA depletion and damage as well as ultrastructural alterations and then cause the alteration in mitochondrial membrane potential (36). The significant decrease of mitochondrial membrane potential implies severe damage of membrane permeability and integrity (36). Mitochondrial permeabilization and dysfunction bring about the release of proapoptotic proteins, subsequently causing cell apoptosis and necrosis (37). The present study showed that the RA pretreatment significantly suppressed the decrease of *∆*Ψ*m* induced by CC1_4_, suggesting a protective effect on mitochondria and an inhibitory effect on apoptosis and necrosis. This finding was highly in agreement with the immunohistochemical result of Caspase-3.

In our previous study, free phenolics from *Lycopus lucidus* Turcz. root (FPLR) with RA predominant possessed significant protective effect on CCl_4_-induced hepatotoxicity in vivo and in vitro (7, 38). Based on the results obtained from the present study, it is reasonable to suggest that RA makes a significant contribution to the hepatoprotective effect of FPLR.

## Conclusions

In conclusion, the results of in vivo experiment indicated that RA exerted a protective effect against CCl_4_-induced liver injury through activating Nrf2 signaling pathway, reducing antioxidant damage, suppressing inflammatory response, and inhibiting hepatocyte apoptosis. Furthermore, the results of in vitro experiment also revealed that RA could attenuate BRL hepatocyte ROS production, DNA oxidative damage, inflammatory response, and apoptosis induced by CCl_4_ exposure. Further research is still imperative to elucidate the activation mechanism of the Nrf2 signaling pathway and anti-inflammatory mechanism of RA in CCl_4_-induced hepatic injury.
